# Supplementary material for: Complexome profiling of the Chlamydomonas psb28 mutant reveals TEF5 as an early PSII assembly factor
Source: Plant Cell. 2025 Mar 17;37(6):koaf055. doi: 10.1093/plcell/koaf055 (PMC12164590; doi:10.1093/plcell/koaf055)
Supplement: koaf055_Supplementary_Data [file koaf055_supplementary_data.zip › psb28_interactive.zip!/plots/intensity/qualityPlots/peptides/CGL152_Cre17.g733950.t1.2.html]

### Cre17.g733950.t1.2

Trivial name: CGL152  
SeqCoverage [median]: 6.20  
Mass: 44.29 kDa  
  
 

| Sequence | MissedCleavage | Mass [Da] | wtIntensity | muIntensity | Score |
| --- | --- | --- | --- | --- | --- |
| LAAQEPR | 0 | 783.42 | 285.65 | 0.00 | 68.79 |
| MAQLSDLFR | 0 | 1079.54 | 0.00 | 436.01 | 101.54 |
|  |  |  | 285.7 | 436.0 |  |
